# How to Increase Post-Thaw Semen Quality in Poor Freezing Stallions: Preliminary Results of the Promising Role of Seminal Plasma Added after Thawing

**DOI:** 10.3390/ani9070414

**Published:** 2019-07-03

**Authors:** Jiří Šichtař, Filipa Bubeníčková, Jitka Sirohi, Ondřej Šimoník

**Affiliations:** 1 Department of Veterinary Sciences, Faculty of Agrobiology, Food and Natural Resources, Czech University of Life Sciences Prague, Kamýcká 129, 165 00 Praha 6–Suchdol, Czech Republic; 2 Department of Statistics, Faculty of Economics and Management, Czech University of Life Sciences Prague, Kamýcká 129, 165 00 Praha 6–Suchdol, Czech Republic

**Keywords:** horse, cryopreservation, semen, freezability

## Abstract

**Simple Summary:**

Approximately half of all stallions are considered poor freezers, which means that they produce ejaculate with poor cryopreservation tolerance. Although the quality of the frozen–thawed semen of poor freezing stallions is low, they can carry and transfer desirable traits to future generations via insemination. Therefore, increasing the post-thaw quality of cryopreserved semen from these stallions is an important step in efficient horse breeding. In this study, we aimed to find a way to increase the quality of cryopreserved semen from poor freezing stallions. We studied the effects of different seminal plasma (SP) additions on various functional parameters of spermatozoa. The results show that the addition of different types of seminal plasma positively affects motility and maintains the status of the plasma membrane of poor freezing stallions. Since the percentage of stallions with poor freezability is quite large, future research should target the discovery of specific seminal plasma components that could be used to increase the post-thaw quality of their spermatozoa.

**Abstract:**

The aim of this study was to evaluate the effect of the addition of two types of seminal plasma (SP) after thawing on the functional characteristics of frozen–thawed (F–T) spermatozoa of poor freezing stallions during prolonged incubation periods. Seminal plasma from stallions with 35–40% (standard seminal plasma, (S-SP)) and 60–70% (above standard seminal plasma, (A-SP)) progressively motile spermatozoa after thawing was used. The motility, kinematic parameters (Computer Assisted Sperm Analysis), distribution of spermatozoa into subpopulations, integrity (carboxyfluorescein diacetate/propidium iodide staining), and functionality (hypo-osmotic swelling (HOS) test) of the spermatozoa plasma membrane were evaluated after thawing (T0) and after 30 min (T30) of incubation at 37 °C. There was no effect of SP addition on spermatozoa motility, but there was a significant positive effect on the kinematic parameters at T0 and T30. The addition of SP significantly increased the percentage of spermatozoa in the fast subpopulation at T0 as well as at T30. Plasma membrane integrity was not affected by the treatment, but functionality significantly decreased by 5% compared to the control group when samples were incubated for 30 min with A-SP. In conclusion, generally, the post-thaw addition of seminal plasma positively affected the post-thaw quality of semen from poor freezing stallions.

## 1. Introduction

Laboratories around the world invest huge effort into increasing the efficiency of semen cryopreservation in stallions [1,2]. Despite this, it is estimated that only 20% of stallions produce semen that can be frozen well and approximately 20–50% produce semen that freezes poorly [3,4]. Since breeding stallions are primarily selected according to their pedigree or performance and not their reproductive efficiency, it is necessary to seek out methods to increase the quality of ejaculate collected from these stallions.

Ejaculate consists of spermatozoa and seminal plasma (SP), with the latter being responsible for the proper protection, introduction, storage, survival, and fertilization ability of spermatozoa in the female reproductive tract during natural mating [5]. Despite these positive effects, almost all seminal plasma is removed from semen before cryopreservation [6] because of its negative effect during processing for preservation or storage [7,8]. On the other hand, efforts have been made to take advantage of this substance in terms of increasing the freezability of spermatozoa [8,9,10].

The influence of seminal plasma on post-thaw spermatozoa characteristics is controversial in various species (as is reviewed in [11]) as well as in good and poor freezing stallions. Some studies have mentioned the beneficial effect on the motility [12], acrosome integrity [13], capacitation status [14], and binding activity of sperm to the zona pellucida in good freezing stallions [10]. On the other hand, other studies failed to find any effect [8,15]. In poor freezing stallions, the addition of seminal plasma was shown to be beneficial for motility, and plasma membrane and acrosome integrity [12]. However, in another study [15], the DNA integrity, mitochondrial membrane potential, plasma membrane integrity, production of reactive oxygen species, and motility remained unchanged when SP was added before cryopreservation.

These inconsistent results are probably related to the different methods of semen processing used (with vs. without the selection of the best spermatozoa), the origin of the SP (heterologous vs. homologous; pooled vs. unpooled), the concentration of SP (5%, 20%, or 30% *v*/*v*), and the time of SP addition (before cryoconservation vs. post-thaw). The origin of seminal plasma seems to be one of the most important factors, as differences in SP composition between individual stallions [16] and good or poor freezers were found [17,18]. Although the addition of SP before storage was shown to increase hydrogen peroxide production and sperm chromatin damage in cooled semen [19], the SP was mostly added before cryopreservation [8,10,12,15]. An exception to this was the study by de Andrade et al. [13], but these authors investigated the effect of SP addition immediately after thawing, which raises the question of whether the effect of such treatment manifests in this timeframe. The manifestation of some effect of SP should be clearer after longer periods of incubation [20]. Moreover, the co-incubation of SP with spermatozoa for longer time intervals better mimics the situation when spermatozoa are deposited into the female reproductive tract during insemination.

Seeing that SP may influence sperm quality and that its addition to frozen–thawed (F–T) semen before insemination may improve the sperm fertilization capability in good freezing stallions [13], we designed a study to evaluate the effects of the addition of two types of seminal plasma after thawing on the functional characteristics of frozen–thawed sperm of poor freezing stallions during prolonged incubation periods.

## 2. Material and Methods

### 2.1. Collection and Processing of Ejaculate

The collection of ejaculate was performed in a certified equine reproductive center (CZ 53790026, Equine Reproduction Centre Ltd., Pardubice-Mnětice, Czech Republic). Sperm-rich fractions were collected in a type of open artificial vagina (n = 10 stallions, 4 collections each). The collected ejaculate was immediately pre-diluted with skim-milk based extender and centrifuged at 650 g for 15 min. Afterwards, the supernatant was removed, and sperm pellets were extended with lactose-EDTA-egg yolk extender, privately manufactured by the Equine Reproduction Centre Ltd., consisting of lactose, distilled water, glycerol, buffers, antibiotics, EDTA, and 20% (*v*/*v*) egg yolk. The final concentration of progressive spermatozoa per mL was 350 × 10^6^.

Extended ejaculate was packed into 5 mL aluminum tubes and horizontally frozen in a Styrofoam box 4 cm above the liquid nitrogen level for 15 min (Animal Reproduction systems, Chino, CA, USA). From each stallion, one insemination dose (ID) from each collection was used for in vitro analysis, resulting in 40 insemination doses. IDs were thawed at 37 °C for 30–60 seconds.

### 2.2. Processing of Seminal Plasma

Standard seminal plasma (S-SP, n = 45) and above-standard seminal plasma (A-SP, n = 45) were obtained from stallions with average post-thaw progressive motilities of 35–40% and 60–70%, respectively. The collection of seminal plasma was performed in two separate collections within a week. Both stallions had previously proven their fertility in the field when frozen–thawed IDs were used for breeding. The average pregnancy rate after insemination with F–T ID was 53% (56 mares inseminated) for the first stallion (S-SP) and 69% (42 mares inseminated) for the second (A-SP) (data obtained from records of Equine Reproduction Centre Ltd.).

To obtain seminal plasma, the gel fraction of the ejaculate was removed and semen was immediately centrifuged at 10,000 × *g* for 10 min. Then, the supernatant was removed and centrifuged under the same conditions again. The absence of spermatozoa in the centrifuged supernatant was confirmed by microscopy. The sperm-free supernatant was frozen at −80 °C until use.

### 2.3. Experimental Design

After thawing, sperm samples were divided into three aliquots, which were diluted either with saline (control group, C) or with S-SP or A-SP. Both SP samples had a concentration of 33.3% *w*/*v*. The qualitative parameters of frozen–thawed IDs were evaluated at T0 (after 5–10 min of co-incubation at 37 °C in a water bath) and T30 (after 30 min of co-incubation).

### 2.4. Evaluation of Sperm Quality Parameters

#### 2.4.1. Motility

Motility was evaluated using a Computer Assisted Sperm Analysis (CASA) system (Nis-Elements, Version 4.50, Laboratory Imaging, Czech Republic). A 3 μL drop of each semen sample was placed in a 37 °C pre-warmed Makler chamber (Sefi-Medical Instrument, Israel), and six fields per sample were evaluated at 100× magnification using a phase-contrast microscope (Eclipse E600; Nikon) equipped with a heating plate pre-warmed at 37 °C.

The evaluation was based on the analysis of 41 consecutive digitized images, which were taken at a time lapse of 0.66 s with a camera at a frequency of 60 fps (DMK 23UM021; Imaging Source, Bremen, Germany). At least 200 trajectories were analyzed per field. The following motility parameters were evaluated: total motility (TMOT, %), progressive motility (PMOT, %), amplitude of lateral head displacement (ALH, μm), beat-cross frequency (BCF, Hz) linearity (LIN, %), straightness coefficient (STR, %), average path velocity (VAP, μm/s), curvilinear velocity (VCL, μm/s), straight line velocity (VSL, μm/s), and wobble (WOB, %). The spermatozoa were considered motile when VAP > 15 μm/s. The threshold values of STR and VAP for progressive motility were 70% and 15 μm/s, respectively. The distribution of spermatozoa into slow, medium fast, and fast subpopulations was based on mean values of BCF, VAP, VCL, and VSL after clustering analysis (see statistical analysis).

#### 2.4.2. Plasma Membrane Integrity (Live/Dead Staining)

To obtain the percentages of live and dead spermatozoa, carboxyfluorescein diacetate (CFDA, Sigma-Aldrich, St. Louis, MO, USA) and propidium iodide (PI, Sigma-Aldrich, USA) staining was used. Briefly, 100 µL of sperm suspension was co-incubated with a solution consisting of 1 µL of 0.3% formaldehyde, 2.1 µL CFDA (0.46 mg/mL of dimethyl sulfoxide), and 2.1 µL PI (0.5 mg/mL of physiological solution). Two smears were prepared per thawed sample and 200 sperm were classified per slide using a fluorescent microscope (Eclipse E600, Nikon, Tokyo, Japan). The green and red cells were classified as live and dead, respectively.

#### 2.4.3. Plasma Membrane Function (HOS Test)

The hypo-osmotic swelling (HOS) test was used to evaluate the functional integrity of the spermatozoa plasma membrane. The spermatozoa suspension (100 µL) was added to hypo-osmotic solution (900 µL), and after 30 min of incubation (37 °C, water bath), the reaction was evaluated in at least 200 cells per sample using a microscope (Eclipse E600, Nikon, Tokyo, Japan). The percentage of swollen spermatozoa (HOS+) was determined, indicating the presence of spermatozoa with a functional and intact plasma membrane.

### 2.5. Statistical Analysis

Statistica software (StatSoft, ver. 12, CZ) was used to evaluate the effect of the addition of the two types of seminal plasma (S-SP, A-SP) on the functional parameters of frozen-thawed stallion spermatozoa. For TMOT, PMOT, plasma membrane integrity (PMI), and HOS testing, the one-way analysis of variance (ANOVA) was used followed by a Tukey test for multiple comparisons.

For the dissection of spermatozoa into subpopulations, the kinematic parameters of spermatozoa were evaluated by k-means cluster analysis. The Euclidean distance algorithm processed BCF, VAP, VCL and VSL with 20 iterations into two clusters (subpopulations) of spermatozoa (slow and fast). A decision tree was used to determine the most important factor affecting the distribution of spermatozoa into subpopulations. A decision tree is a data mining method used to find any interesting unknown patterns in a large database and can classify objects or instances into a predefined set of classes based on values, in this experiment, the time or type of seminal plasma. In decision trees, groups are created that become more homogeneous, thereby representing different classes into which the objects are classified [21]. The χ^2^ test was used to determine differences between subpopulations [22]. The TMOT, PMOT, PMI, and HOS, and subpopulations were evaluated at *P* < 0.05, and CASA parameters were evaluated at *P* < 0.01. Unless otherwise indicated, the data are presented as LSM ± SEM.

## 3. Results

### 3.1. Effect of Different Types of Seminal Plasma on Spermatozoa Motility and Kinematic Parameters

The motility characteristics of the treatments are shown in Table 1. All stallions included in this study were characterized as poor freezers as they produced IDs with ≤20% of progressively motile spermatozoa post-thaw. The addition of seminal plasma did not alter the total and progressive motility (*P* > 0.05) at T0 or T30. Thirty minutes of incubation led to decreases in TMOT and PMOT (*P* < 0.05).

The addition of two types of seminal plasma significantly influenced CASA parameters at both T0 and T30 (*P* < 0.01) (Table 1). Incubation of the frozen–thawed spermatozoa of poor freezing stallions with seminal plasma for 30 min increased five out of the seven studied CASA parameters, specifically LIN, STR, VAP, VSL, and WOB, compared to the control group (*P* < 0.01). CASA parameters that showed significant differences between S-SP and A-SP were ALH and STR (*P* < 0.01), and significantly higher values compared to the C group were found in LIN (both S-SP and A-SP), STR (A-SP), VAP (S-SP), VSL (S-SP), and WOB (both S-SP and A-SP) (*P* < 0.01).

### 3.2. Effect of Different Types of Seminal Plasma on the Subpopulation Distribution of Spermatozoa

**Figure 1:**
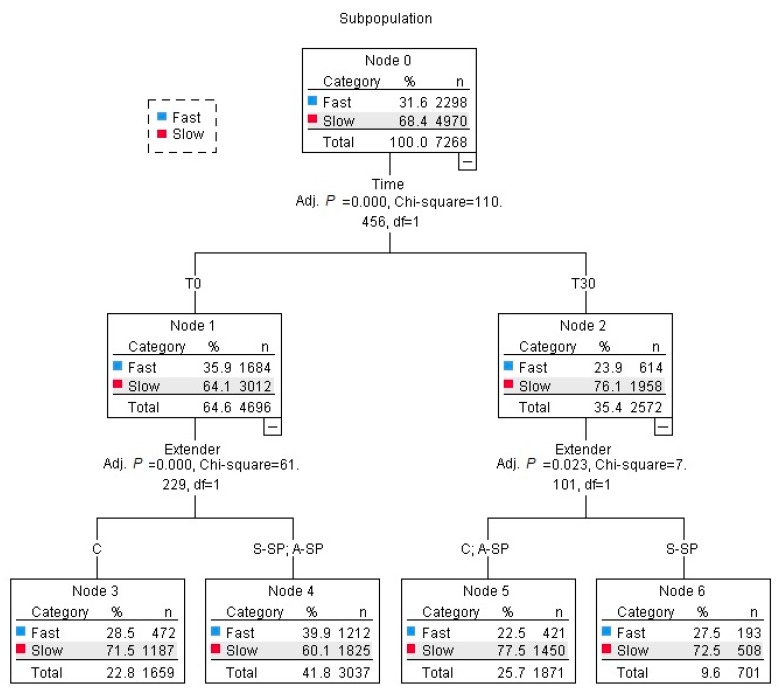
Effect of seminal plasma addition on the distribution of spermatozoa into slow and fast subpopulations after thawing and after 30 min of incubation.

The distribution of spermatozoa to subpopulations (fast and slow) was significantly affected by the incubation time and the type of SP added (*P* < 0.05) (Figure 1). The addition of S-SP and A-SP yielded more spermatozoa in the fast subpopulations compared to the control (*P* < 0.05). After 30 min of incubation, the highest percentage of spermatozoa was achieved in the fast subpopulations where S-SP was added compared to in the A-SP and C samples (*P* < 0.05).

### 3.3. Effect of Different Types of Seminal Plasma on Plasma Membrane Status

The results of plasma membrane integrity (PMI) and functionality (HOS+) are shown in Table 2. The addition of seminal plasma, regardless of its origin, did not alter the PMI at T0 or T30 (*P* > 0.05). The percentage of HOS+ spermatozoa was not affected by SP treatment at T0 but decreased at T30 in A-SP samples compared to the control group (*P* < 0.05). Incubation for 30 min significantly decreased the PMI in all treatment groups (*P* < 0.05); however, the percentage of HOS+ spermatozoa did not change during the incubation period (*P* > 0.05).

## 4. Discussion

The results of this study show that the post-thaw addition of seminal plasma to poor freezing stallion sperm from stallions with average and above average freezability has beneficial effects on the kinematic parameters of the sperm and the distribution of spermatozoa to subpopulations. The seminal plasma used in our study was not pooled, as it was in other studies [10,12]; hence, the effects of standard and above-standard SP could be evaluated more precisely. Moreover, our study evaluated the function of SP on F–T spermatozoa over a prolonged time interval to better mimic the post-insemination situation in the female reproductive tract.

Our results show that the addition of SP from both average and above-average freezable stallions failed to influence the total and progressive motility of spermatozoa in poor freezing stallions when added after thawing. Similarly, de Andrade et al. [13] found no effect on total motility when homologous or autologous SP was added from good freezers after the thawing of semen. However, progressive motility significantly decreased after the addition of autologous SP. Although SP activates the motility of epididymal spermatozoa [23], it seems that this physiological function does not always affect ejaculated spermatozoa in the same way when SP is added [8,10,12,15]. The most likely reason for this is the variability in the SP components among individual stallions [9], and particularly, between good and poor freezers [18]. Moreover, the motility stimulant aminopeptidase N differs between the seminal plasma of good and poor freezers [15].

Even though subjective motility evaluation is the gold standard under field conditions, the use of CASA systems, as was done in the current study, is highly recommended to reveal the more subtle changes in spermatozoa [24]. With this approach, we demonstrated that the addition of SP (both S-SP and A-SP) after thawing had a positive effect on several kinematic parameters (ALH, LIN, STR, VAP, VCL, VSL, and WOB) in poor freezing stallions. Interestingly, the results of a study by de Andrade et al. [13] showed that the addition of autologous SP from good freezing stallions to semen after thawing only increased ALH. On the other hand, Neuhauser et al. [25] published increased CASA parameters when autologous SP was added after thawing. In our experiment, the subsequent 30 min incubation of semen with both S-SP and A-SP benefitted the kinematic parameters of spermatozoa.

In addition to the mentioned effect on kinematic parameters, we also, as with the first study, found a positive effect of the addition of SP on the distribution of spermatozoa into subpopulations. The existence of various subpopulations of spermatozoa in ejaculate is well known, see for example [26,27], and the simple averaging of kinematic parameters may lead to misleading results. Despite this fact, this approach is still lacking/ignored in many studies dealing with specific effects on spermatozoa motility.

In the conditions of our study, we found that the addition of S-SP positively affected the distribution of spermatozoa in fast subpopulations after 30 min of incubation. In bovine models, the percentage of spermatozoa in fast subpopulations was strongly correlated with in vitro fertilization results (zona binding, penetration rate, and pronucleus formation) [28]. Moreover, since the speed of spermatozoa is based on these parameters with a correlation with fertility per cycle [29], the results of our study may imply that the addition of SP to frozen–thawed spermatozoa from poor freezing stallions increases the fertilizing potential of their F–T semen.

The plasma membrane of spermatozoa plays an important role in several processes that lead to the successful fertilization of the oocyte [30]. Seminal plasma contains factors (mainly proteins) that affect the status of the plasma membrane of spermatozoa, regulating the onset of the capacitation and acrosome reaction [31]. In our study, the addition of S-SP and A-SP to F–T semen from poor freezing stallions failed to affect sperm membrane integrity. In the case of A-SP addition, the functionality of the plasma membrane decreased by approximately 5% after 30 min of incubation. This is in contrast with results in good freezing stallions, where the functionality of the plasma membrane significantly increased when homologous or autologous SP was added, although that effect was only evaluated immediately after thawing [13].

Seminal plasma components bind to spermatozoa during their route through a stallion’s reproductive tract. Hence, the development of spermatozoa is complete before the semen is manipulated for freezing. As the seminal plasma components differ between good and poor freezers [18], we can assume that collected ejaculate from poor freezers has predetermined freezability. This is further supported by a study by Cabrera et al. [32], who found different lipidomic profiles in poor compared to good freezers and linked this change to freezability.

This different profile in poor freezers is probably related to a decreased ability to withstand osmotic challenges during temperature changes [4]. The representation of lipids and the cholesterol to lipid ratio in plasma membrane plays a crucial role in maintaining membrane fluidity, which is related to the susceptibility to damage caused by low temperatures [33], and the decrease of temperature is, of course, connected with the cooling of semen during successful cryopreservation [34].

## 5. Conclusions

In conclusion, the post-thaw addition of seminal plasma from average and above-average freezable stallions positively affects sperm motility and maintains the plasma membrane status of poor freezing stallions following a 30 min post-thaw incubation period. Although, generally, the ID from poor freezers contains less motile spermatozoa compared to good freezers, the frozen–thawed semen could be used via deep horn insemination or for the in vitro production of equine embryos where selection of the best spermatozoa is performed.

Since there are discrepancies among the results of studies describing the effect of seminal plasma addition to equine frozen spermatozoa, future research should focus on more detailed study of seminal plasma and its components. There is increasing evidence that some components of SP may play positive roles and others may play negative roles in this process. The isolation of these positive substances and the testing of their effects on frozen–thawed spermatozoa, especially when poor freezers need to produce F–T IDs, may reveal new cryopreservation strategies for ejaculate from these stallions.

## Figures and Tables

**Table 1:** Total motility (TMOT), progressive motility (PMOT), and kinematic parameters of spermatozoa after thawing (T0) and after 30 min incubation at 37 °C (T30) with the addition of seminal plasma.

Motility and Kinematic Parameters	Time (min.)	C	S-SP	A-SP
TMOT (%)	T0	13.5 ± 1.2 ^a^	13.4 ± 1.2 ^a^	13.1 ± 1.2 ^a^
	T30	10.8 ± 1.0 ^b^	8.2 ± 0.9 ^b^	10.1 ± 0.9 ^b^
PMOT (%)	T0	11.1 ± 1.2 ^a^	12.0 ± 1.2 ^a^	11.9 ± 1.2 ^a^
	T30	9.0 ± 0.8 ^b^	7.9 ± 0.8 ^b^	9.1 ± 0.8 ^b^
ALH (µm)	T0	3.4 ± 0.1 ^1^	3.9 ± 0.1 ^2 a^	3.7 ± 0.1 ^a 3^
	T30	3.4 ± 0.1	3.6 ± 0.1 ^1 b^	3.3 ± 0.1 ^b 2^
BCF (Hz)	T0	11.0 ± 0.2 ^a^	11.3 ± 0.2 ^a^	11.6 ± 0.2 ^a^
	T30	9.6 ± 0.2 ^b^	10.2 ± 0.2 ^b^	10.3 ± 0.2 ^b^
LIN (%)	T0	38.5 ± 0.3 ^a 1^	40.1 ± 0.4 ^a 2^	40.7 ± 0.3 ^a 2^
	T30	33.3 ± 0.4 ^b 1^	36.0 ± 0.5 ^b 2^	37.2 ± 0.5 ^b 2^
STR (%)	T0	85.7 ± 0.4 ^a 1^	85.9 ± 0.4^a 1^	87.6 ± 0.4 ^2^
	T30	80.8 ± 0.4 ^b 1^	82.3 ± 0.6 ^b 1^	85.6 ± 0.6 ^2^
VAP (µm/s)	T0	42.4 ± 0.6 ^a 1^	50.1 ± 0.7 ^a 2^	48.7 ± 0.6 ^a 2^
	T30	37.1 ± 0.7 ^b 1^	40.8 ± 1.0 ^b 2^	38.2 ± 1.0 ^b^
VCL (µm/s)	T0	95.9 ± 1.3 ^1^	109.8 ± 1.4 ^a 2^	106.5 ± 1.3 ^a 2^
	T30	92.4 ± 1.5	96.1 ± 1.9b	88.9 ± 2.0 ^b^
VSL (µm/s)	T0	37.4 ± 0.6 ^a 1^	44.5 ± 0.7 ^a 2^	44.0 ± 0.7 ^a 2^
	T30	31.0 ± 0.7 ^b 1^	34.8 ± 0.9 ^b 2^	33.9 ± 0.9 ^b^
WOB (%)	T0	44.3 ± 0.3 ^a 1^	45.8 ± 0.3 ^a 2^	45.8 ± 0.3 ^a 2^
	T30	40.7 ± 0.3 ^b 1^	43.1 ± 0.4 ^b 2^	42.8 ± 0.4 ^b 2^

^a,b^ data with different superscripts within a column significantly differ between T0 and T30 ^1,2^ data with different superscripts within a row significantly differ between experimental groups C (control group), S-SP (standard SP), and A-SP (above-standard SP). ALH: amplitude of lateral head displacement; BCF: beat-cross frequency; LIN: linearity; STR: straightness coefficient; VAP: average path velocity; VCL: curvilinear velocity; VSL: straight line velocity; WOB: wobble.

**Table 2:** Viability and plasma membrane integrity (HOS+) after thawing (T0) and after an incubation time of 30 min (T30): C (control group), S-SP (standard Sp), A-SP (above-standard SP).

Treatment	PMI (%)		HOS+ (%)	
	T0	T30	T0	T30
C	38.5 ± 1.6^a^	29.3 ± 1.7^b^	25.0 ± 2.0	24.2 ± 1.6^1^
S-SP	37.8 ± 1.7^a^	29.2 ± 1.9^b^	23.6 ± 2.1	22.9 ± 1.8
A-SP	37.2 ± 1.7^a^	33.0 ± 1.9^b^	24.5 ± 2.1	19.4 ± 1.8^2^

^a,b^ data with different superscripts within a row significantly differ; ^1,2^ data with different superscripts within a column significantly differ. PMI: plasma membrane integrity; HOS: hypo-osmotic swelling.
